# Identification and Validation of miR-222-3p and miR-409-3p as Plasma Biomarkers in Gestational Diabetes Mellitus Sharing Validated Target Genes Involved in Metabolic Homeostasis

**DOI:** 10.3390/ijms23084276

**Published:** 2022-04-12

**Authors:** Tiziana Filardi, Giuseppina Catanzaro, Giuseppina Emanuela Grieco, Elena Splendiani, Sofia Trocchianesi, Carmela Santangelo, Roberto Brunelli, Elisa Guarino, Guido Sebastiani, Francesco Dotta, Susanna Morano, Elisabetta Ferretti

**Affiliations:** 1 Department of Experimental Medicine, “Sapienza” University, 00161 Rome, Italy; tiziana.filardi@uniroma1.it (T.F.); susanna.morano@uniroma1.it (S.M.); elisabetta.ferretti@uniroma1.it (E.F.); 2 Diabetes Unit, Department of Medicine, Surgery and Neurosciences, University of Siena, 53100 Siena, Italy; giusy.grieco90@gmail.com (G.E.G.); sebastianiguido@gmail.com (G.S.); francesco.dotta@unisi.it (F.D.); 3 Fondazione Umberto di Mario, Toscana Life Sciences, 53100 Siena, Italy; 4 Department of Molecular Medicine, “Sapienza” University, 00161 Rome, Italy; elena.splendiani@uniroma1.it (E.S.); sofia.trocchianesi@uniroma1.it (S.T.); 5 Center for Gender-Specific Medicine, Gender Specific Prevention and Health Unit, Istituto Superiore di Sanità, 00161 Rome, Italy; carmela.santangelo@iss.it; 6 Maternal and Child Health and Urological Sciences, “Sapienza” University, 00161 Rome, Italy; roberto.brunelli@uniroma1.it; 7 UOC Diabetologia, Azienda Ospedaliera Universitaria Senese, 53100 Siena, Italy; eguarino70@gmail.com; 8 Tuscany Centre for Precision Medicine (CReMeP), 53100 Siena, Italy

**Keywords:** gestational diabetes mellitus, GDM, miRNA, miR-222-3p, miR-409-3p, extracellular vesicles, GDM pathophysiology, pregnancy complications

## Abstract

Gestational diabetes mellitus (GDM) causes both maternal and fetal adverse outcomes. The deregulation of microRNAs (miRNAs) in GDM suggests their involvement in GDM pathogenesis and complications. Exosomes are extracellular vesicles (EVs) of endosomal origin, released via exocytosis into the extracellular compartment. Through EVs, miRNAs are delivered in distant target cells and are able to affect gene expression. In this study, miRNA expression was analyzed to find new miRNAs that could improve GDM classification and molecular characterization. MiRNA were profiled in total plasma and EVs in GDM patients and normal glucose tolerance (NGT) women. Samples were collected at third trimester of gestation from two diabetes centers. MiRNA expression was profiled in a discovery cohort using the multiplexed NanoString nCounter Human v3 miRNA. Validation analysis was performed in a second independent cohort using RT-qPCR. A set of miRNAs resulted to be differentially expressed (DE) in total plasma and EVs in GDM. Among them, total plasma miR-222-3p and miR-409-3p were validated in the independent cohort. MiR-222-3p levels correlated with fasting plasma glucose (FPG) (*p* < 0.001) and birth weight (*p* = 0.012), whereas miR-409-3p expression correlated with FPG (*p* < 0.001) and inversely with gestational age (*p* = 0.001). The major validated target genes of the deregulated miRNAs were consistently linked to type 2 diabetes and GDM pathophysiology. MiR-222-3p and miR-409-3p are two circulating biomarkers that could improve GDM classification power and act in the context of the molecular events leading to the metabolic alterations observed in GDM.

## 1. Introduction

Gestational Diabetes Mellitus (GDM) is a metabolic disease diagnosed in the second or third trimester of pregnancy [1]. The prevalence of this condition is reaching alarming numbers worldwide, due to the large spread of obesity [2,3]. Unless properly diagnosed and treated, GDM can lead to poor pregnancy outcomes [4,5,6,7,8,9].

The pathophysiological features of GDM mainly include maternal insulin resistance, β-cell dysfunction, placental dysfunction, endothelial dysfunction, and inflammation [10,11,12,13,14]. However, the molecular mechanisms involved in the onset of this condition and related complications are not yet fully uncovered.

MicroRNAs (miRNAs) are small non-coding RNAs that act as destabilizing or depleting target mRNAs [15]. Their dysregulation has been described in the context of numerous pathological processes, including metabolic diseases. A growing number of studies have characterized miRNA expression in biological fluids and in gestational tissues, highlighting their crucial involvement both in the pathogenic mechanisms of GDM and in the development of GDM-related short- and long-term complications [16]. Specifically, several studies identified a wide number of differentially expressed (DE) tissue miRNAs critically involved in energy control, lipid homeostasis and inflammation, playing putative roles in the development of insulin resistance in pregnancy [17,18,19,20,21,22]. Recent studies have reported a possible miRNA-mediated cross-talk between placenta and maternal β-cells [23,24,25,26]. Moreover, a complex link between several dysregulated miRNAs and impairment in placenta development and functions in GDM has been described, contributing to adverse outcomes [27,28,29]. Indeed, miRNAs seem to play crucial roles in regulating foetal growth [30,31]. Besides short-term complications, miRNA deregulation in pregnancy might contribute to the development of long-term GDM-related complications, such as metabolic and cardiovascular diseases, both in the mother and in the offspring [32,33,34,35,36].

Early diagnosis and appropriate treatment (lifestyle intervention or insulin therapy) are mandatory to avoid poor pregnancy outcomes in GDM. The current diagnostic criteria for GDM have been established by the International Association of Diabetes and Pregnancy Study Groups (IADPSG) [4]. The IADPSG recommends performing an oral glucose tolerance test (OGTT) with 75 g-glucose at 24–28 weeks of gestation. Fasting, 1-h and 2-h plasma glucose cut-off values for GDM diagnosis are 5.1 mmol/L, 10 mmol/L, and 8.5 mmol/L, respectively [37]. However, novel circulating biomarkers that can help in diagnosing GDM earlier during gestation might reduce the incidence of maternal and foetal complications. Since miRNAs can be potentially isolated from all body fluids, they are candidate biomarkers for several pathological conditions, including GDM [38]. In light of this, several studies have investigated the putative role of miRNAs as circulating plasma/serum biomarkers for GDM [16]. Some of them have focused on the expression of miRNAs in exosomes, extracellular vesicles (EVs) of endosomal origin released via exocytosis into the extracellular compartment [39]. EVs are known to control a wide variety of cellular functions in target cells, namely protein synthesis, proliferation, apoptosis, angiogenesis, and metabolic functions [40]. EVs contain small RNA molecules, including mRNAs and miRNAs, which are therefore protected from RNAse digestion [38]. Thus, through EVs, miRNAs are delivered in target cells and are able to regulate gene expression. Plasma EV concentration is significantly increased in GDM pregnancies compared to healthy ones, and a high glucose concentration is able to induce exosome delivery by trophoblast cells [38,41]. In particular, a two-fold higher plasma exosome concentration has been observed throughout GDM pregnancies compared with normal pregnancies, speculating a conceivable role of plasma exosome profiling in predicting GDM at 11–14 weeks, long before the time of the actually recommended screening (24–28 weeks) [42]. Different patterns of expression of miRNA in exosomes isolated from chorionic villi, muscle biopsies, breast milk, and urine have been observed in GDM compared to control subjects [21,43,44]. However, to date, evidence about miRNA profiling in circulating EVs in GDM is limited.

The aim of this study was to evaluate the miRNA expression profile both in total plasma and circulating EVs of patients with GDM in the third trimester of gestation to identify novel biomarkers for GDM and define their involvement in disease pathophysiology.

## 2. Results

### 2.1. Clinical and Biochemical Characteristics of the Discovery and the Validation Cohort

A discovery cohort of *n* = 3 GDM patients (mean age: 34.7 ± 4.9 years; BMI 27.0 ± 3.7 Kg/m^2^) and *n* = 3 pregnant women with normal glucose tolerance (NGT) (mean age: 34.3 ± 3.1 years; BMI 26.4 ± 1.1 Kg/m^2^), and a validation cohort of *n* = 12 GDM patients (mean age: 36.4 ± 4.6 years; BMI: 25.8 ± 3.8 Kg/m^2^) and *n* = 12 NGT women (mean age: 34.9 ± 5.1 years; BMI: 22.4 ± 3.2 Kg/m^2^) were recruited. The clinical and biochemical characteristics of the discovery and the validation cohorts are reported in Table 1 and Table 2, respectively.

### 2.2. MiRNA Profiling and Enrichment Pathway Analysis

First of all, isolated EVs were analyzed for their purity. As shown in Supplementary Figure S1, EVs were positive for Hsp70, CD63 and Tsg101, and were negative for Calnexin. These results confirm that EVs were pure and successfully isolated.

**Figure 1:**
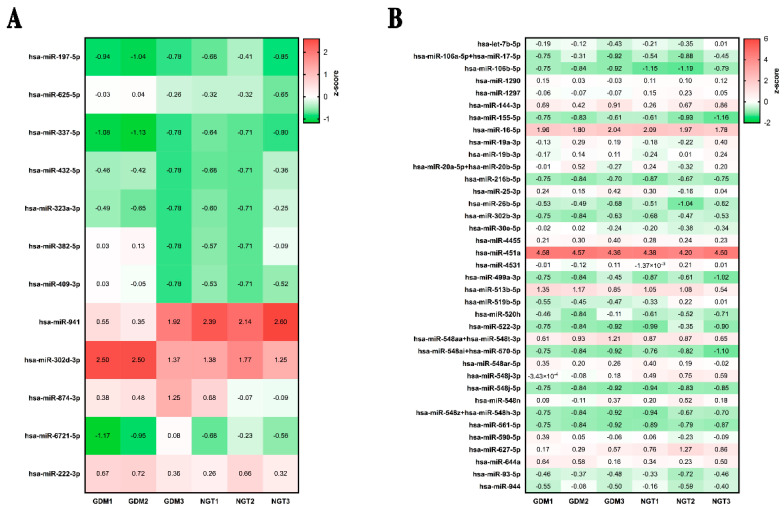
Analysis of differentially expressed (DE) miRNAs in GDM vs. NGT patients of the discovery cohort. Hierarchical clustering analysis of the DE miRNAs in total plasma (**A**) and EVs (**B**) of GDM (*n* = 3) and NGT (*n* = 3) patients demonstrates the miRNAs different expression pattern between the two subgroups.

All RNA samples were profiled with the NanoString nCounter Human v3 miRNA expression assay. A total number of 800 human miRNAs were analyzed. The number of detectable miRNAs was significantly higher in total plasma than in EVs samples (Data not shown). Specifically, in total plasma, an average of 222 and 204 miRNAs were detected in GDM patients and NGT subjects, respectively. Conversely, the number of detectable miRNAs in EVs samples was 75 in GDM patients and 93 in NGT subjects. The comparison between GDM and NGT pregnant women allowed to detect 12 DE miRNAs in total plasma, 11 upregulated and 1 downregulated (Supplementary Table S2), and 37 downregulated miRNAs in the EVs (Supplementary Table S3). The total plasma and EV DE miRNAs allowed to clearly distinguish the two groups of patients, as reported in the hierarchical clustering analysis (Figure 1A,B).

**Figure 2:**
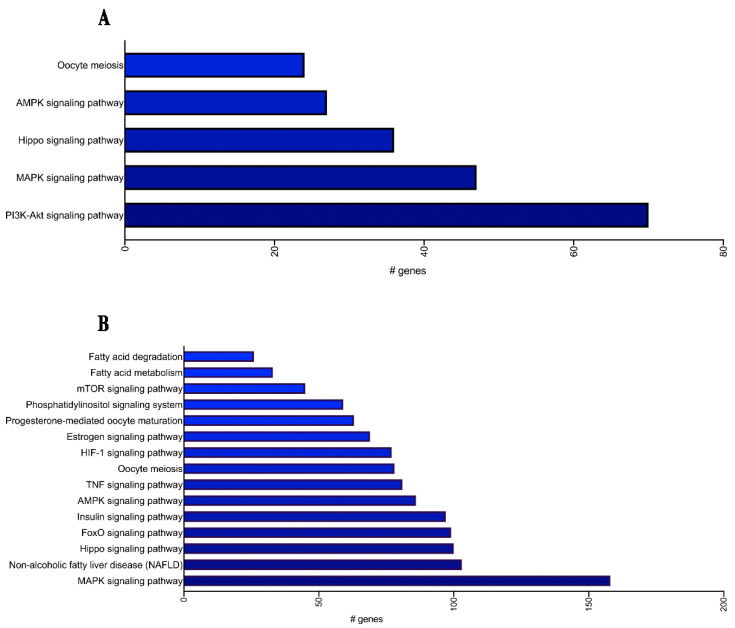
Enrichment analysis of differentially expressed (DE) miRNAs in GDM vs. NGT patients of the discovery cohort. Enrichment analysis of DE total plasma (**A**) and EVs-miRNAs (**B**) was performed with KEGG and demonstrates the involvement of a number of DE total plasma and EVs-miRNAs in GDM signaling pathways.

The biological relevance of these miRNAs was then investigated by performing enrichment pathway analysis and, for both total plasma and EV DE miRNAs, an involvement in GDM-related pathways was uncovered, as shown in Figure 2A,B.

In particular, 7 out of the 12 DE miRNAs detected in total plasma and 12 out of the 37 DE miRNAs detected in EVs (Table 3) were found to be involved in key pathways implicated in GDM and type 2 diabetes (T2D) pathophysiology, such as the insulin signaling pathway, AMP-activated protein kinase (AMPK), phosphatidylinositol-3 kinase (PI3K)/protein kinase B (AKT) and Forkhead box protein O (FoxO) signaling pathway.

The miRNAs involved in GDM pathways were considered as the most biological relevant DE miRNAs. Among them, the ones whose relevant GDM-related experimentally validated target genes showed concordant expression in the presence or in the absence of T2D, according to literature, were selected for validation analysis.

### 2.3. MiRNA Validation in an Independent Cohort and Correlation Analyses

**Figure 3:**
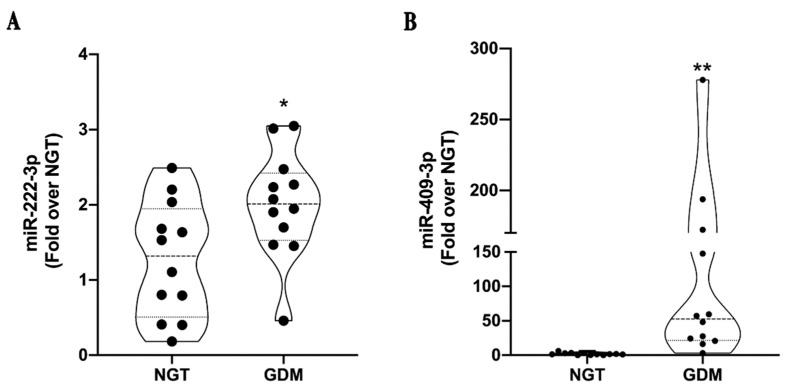
RT-qPCR analysis of miR-222-3p and miR-409 in the validation cohort. RT-qPCR was used to analyze miR-222-3p (**A**) and miR-409-3p (**B**) in the total plasma compartment of the GDM (*n* = 12) and NGT (*n* = 12) validation cohort. The analysis confirmed the upregulation of these two miRNAs in GDM patients vs. NGT subjects. Significance was calculated with GraphPad Prism software by using the two-tailed t-test and calculating the difference between the mean of GDM vs. NGT ± standard error of the mean (SEM). * *p* < 0.05, ** *p* < 0.01.

For validation studies, the miRNAs selected from the discovery cohort profiling (Table 3) were validated in a larger cohort. The samples of the validation cohort were analyzed for both total plasma and EV miRNAs. Two miRNAs, namely miR-222-3p and miR-409-3p, in the total plasma compartment confirmed their upregulation in GDM patients in respect of NGT pregnant women (Figure 3A,B).

**Figure 4:**
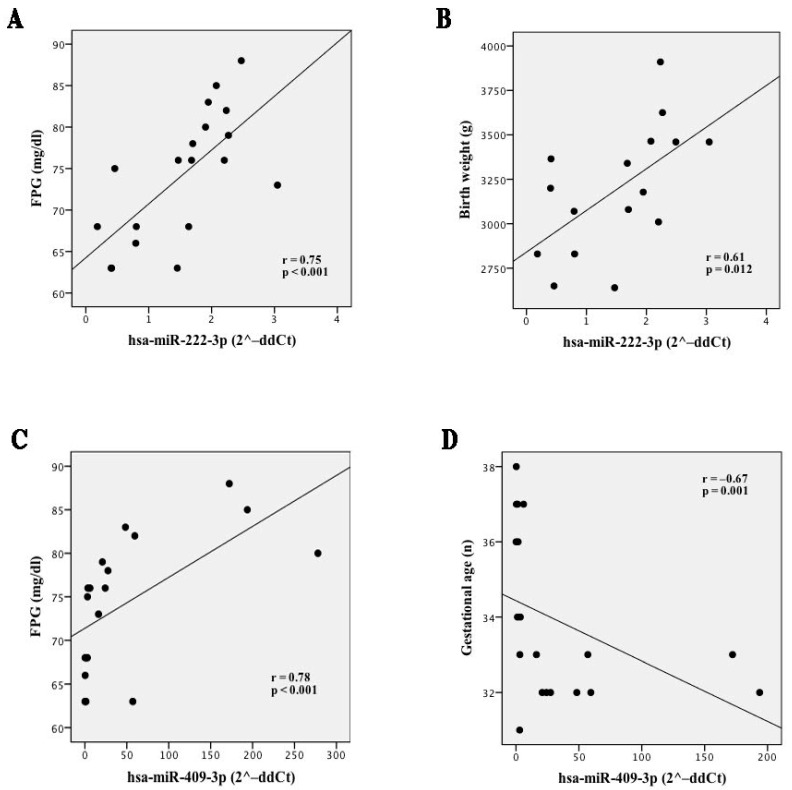
Correlation analyses for miR-222-3p and miR-409-3p with clinical variables. Correlation analyses between miR-222-3p and FPG (**A**) hsa-miR-222-3p and birth weight (**B**); hsa-miR-409-3p and FPG (**C**); and hsa-miR-409-3p and gestational age (**D**). Correlation analysis was performed in 24 samples (GDM, *n* = 12; NGT *n* = 12). Expression levels are reported as normalized 2^–ddCt^ values. Spearman R test was performed to evaluate *r*-values and *p*-values (*p* < 0.05).

Interestingly, the expression of circulating miR-222-3p was positively correlated to fasting plasma glucose (FPG) (*p* < 0.001) and birth weight (*p* = 0.012) (Figure 4A,B), whereas the expression of circulating miR-409-3p was significantly directly correlated to FPG (*p* < 0.001) and inversely correlated with gestational age (*p* = 0.001) (Figure 4C,D).

Of note, none of the 12 EVs miRNAs selected for validation studies confirmed its downregulation; this result can be due to fact that the number of detectable miRNAs was significantly lower in EVs in respect to total plasma compartment, suggesting that EVs miRNAs are released in very low amounts in these samples and rendering the selection of candidate biomarkers harder.

### 2.4. MiR-222-3p and miR-409-3p Target Genes Are Involved in Glucose Homeostasis and T2D

**Figure 5:**
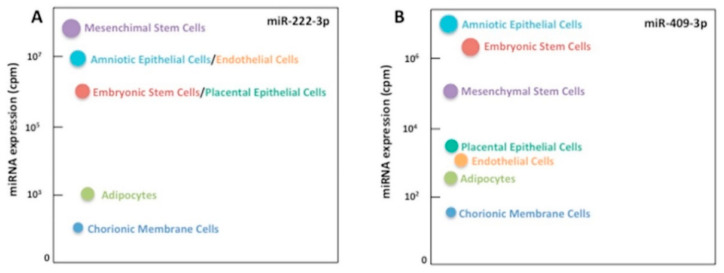
Expression of miR-222-3p and miR-409-3p in specific primary cell samples. Expression of miR-222-3p (**A**) and miR-409-3p (**B**) was enriched in mesenchymal stem cells, amniotic and placental epithelial cells, endothelial cells, and embryonic stem cells; lower expression of these miRNAs was found in adipocytes and chorionic membrane cells. cpm = counts per million.

An in-silico analysis with the evaluation of the expression of miR-222-3p and miR-409-3p in different cell types was performed. These miRNAs are highly expressed in mesenchymal stem cells and also enriched in pregnancy-related cell types, such as embryonic stem cells, amniotic epithelial cells, and placental epithelial cells (Figure 5A,B and Supplementary Tables S4 and S5). 

In addition, miR-222-3p and miR-409-3p share four experimentally validated target genes, namely the serine/threonine-protein phosphatase 2A 55 kDa regulatory subunit B alpha isoform (PPP2R2A); vestigial-like family member 4 (VGLL4); reversion-inducing cysteine rich protein with kazal motifs (RECK); and O-6-methylguanine-DNA methyltransferase (MGMT) and, with the exception of VGLL4, low levels of all these genes have been related with T2D, as reported in Table 4. Interestingly, in healthy subjects, these molecules resulted to be highly expressed in the female reproductive tract.

**Figure 6:**
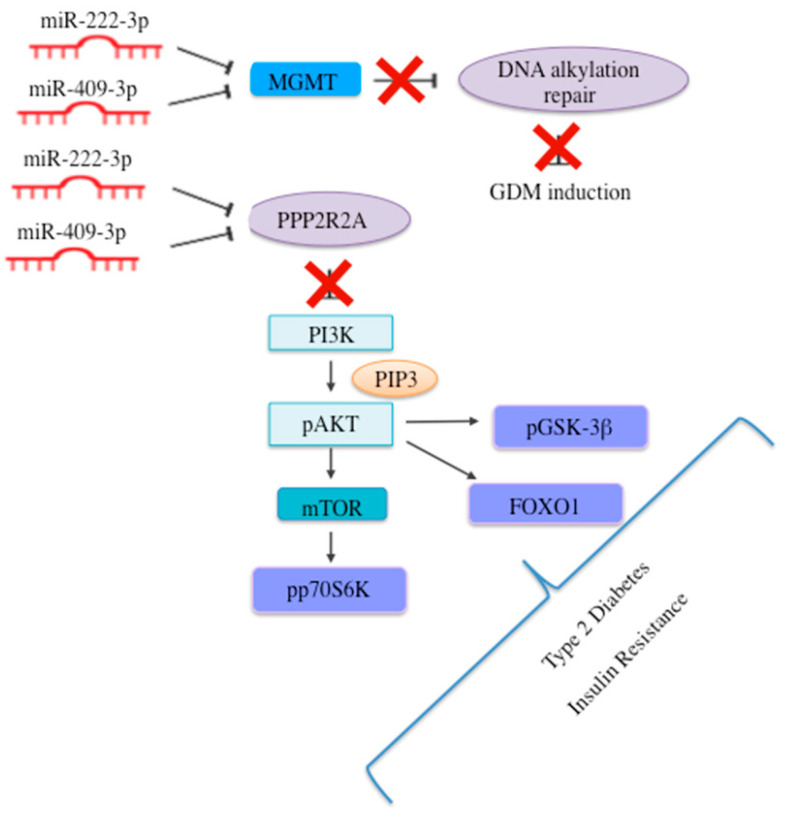
Hypothesis of the role of miR-222-3p and miR-409-3p and their validated targets in the pathophysiology of GDM.

The overview of literature data and of the expression of miR-222-3p and miR-409-3p together with their validated targets allowed to hypothesize the putative role of these miRNAs in the pathophysiology of GDM, as summarized in Figure 6.

## 3. Discussion

Several studies have previously analyzed miRNA expression profile in the plasma and serum of patients with GDM adopting high-throughput techniques, leading to conflicting results [16]. The inconsistencies might be explained by the differences in the main characteristics of the enrolled samples or in the analytical methods employed for miRNA expression profiling. Of note, miRNA expression has been shown to differ among various stages of pregnancy, as well as between serum and plasma [48,49].

In our study, miRNA expression has been profiled in total plasma and EVs isolated from women with GDM and NGT at the third trimester of pregnancy. The different patterns of expression between the two groups suggested that GDM pregnancy might be hallmarked by profound miRNA changes.

The validation analysis performed in a larger cohort of GDM and NGT allowed to validate two upregulated total plasma, miR-222-3p and miR-409-3p. In previous studies, miR-222-3p has been consistently linked to GDM. However, its role has not yet been clarified. Tagoma et al. reported the upregulation of miR-222-3p in plasma derived from GDM patients in comparison with healthy subjects [50]. Conversely, Zhao et al. reported the opposite result [51]. However, Zhao et al. analyzed serum miRNAs at an earlier stage of gestation (16–19 weeks), when the metabolic changes have not completely developed yet. Moreover, Pheiffer et al. reported a significant reduction of serum miR-222-3p in GDM patients compared to healthy pregnant women [52]. MiRNA expression has been shown to vary between serum and plasma samples, as well as among different trimesters of pregnancy [48,49], possibly explaining the discrepancy between these results. Notably, miR-222 cluster has been reported to be particularly abundant in maternal plasma throughout pregnancy, with peak expression during 24–28 gestational weeks [48]. MiR-222 expression has been previously evaluated at a tissue level as well, in GDM. For instance, Shi et al. evaluated the miRNA expression profile in omental visceral adipose tissue (VAT) from GDM and healthy pregnant women at caesarean delivery, identifying an upregulation of miR-222 in GDM [53]. It is worth noting that miR-222 levels were negatively correlated with estrogen receptor (ER)-α and GLUT4. Additionally, GDM women showed increased levels of serum estradiol in respect to healthy ones [53]. Moreover, 3T3-L1 adipocytes were used to functionally validate the direct targeting of miR-222 on ER-α [53]. Estradiol and ER-α both act on GLUT4 and represent critical regulators of obesity and insulin resistance [54,55,56], therefore, these results highlight the importance of miR-222 as a modulator of the expression of ER-α in estrogen-induced insulin resistance. Interestingly, in our study, the expression of circulating miR-222-3p was significantly and positively correlated both to plasma glucose and birth weight, suggesting a potential link between this miRNA and glucose metabolism in pregnancy, which is known to deeply influence birth weight.

For the first time herein, the expression of circulating miR-409-3p has been evaluated in GDM. In particular, miR-409-3p was significantly overexpressed in GDM vs. NGT, and a positive correlation with FPG was observed. Recently, miR-409-3p emerged as a regulator of tissue crosstalk between endothelial cells and brown adipose tissue in mice [57]. In another study, miR-409-3p was upregulated in VAT obtained from patients with impaired fasting glucose, compared to NGT [58]. In addition, in a previous study, miR-409-5p was linked to GDM, displaying higher serum expression in GDM patients compared to the control group [59]. A significant positive correlation between miR-409-5p expression and FPG, HbA1c, and Homeostasis Model Assessment of Insulin Resistance (HOMA-IR) has also been described, suggesting that the miR-409 family might be involved in glucose homeostasis [59]. Of note, in this study, a negative correlation between miR-409-3p and gestational age has emerged, suggesting possible changes in the expression of this miRNA according to different stages of pregnancy.

In-silico analysis miR-222-3p and miR-409-3p putative cell origin resulted in embryonic stem cells, amniotic epithelial cells, and placental epithelial cells. Furthermore, validated target genes of miR-222-3p and miR-409-3p include PPP2R2A, RECK, and MGMT, all implicated in T2D and GDM pathophysiology [45,46,47]. Specifically, PPP2R2A belongs to the PI3K/AKT pathway, which has a well-established role in glucose and lipid metabolism [60]. Accordingly, other experimentally validated target genes of miR-222-3p or miR-409-3p, such as AKT3, insulin receptor substrate 4 (IRS4), Phosphatase and Tensin Homolog (PTEN), and AKT1, belong to the PI3K/AKT pathway. Overall, these findings suggest that these miRNAs might be involved in pregnancy-specific deregulation of the PI3K-AKT pathway.

In conclusion, we reported two new circulating biomarkers, miR-222-3p and miR-409-3p, which alone or in combination with other biomarkers could improve GDM classification power and help understand the pathophysiology of GDM and related complications. The role of the two miRNAs as useful biomarkers of GDM is supported by the fact that their validated target genes are involved in the metabolic alterations observed in GDM.

## 4. Materials and Methods

### 4.1. Patients

GDM patients were recruited in two Italian diabetes centers, specifically the Diabetes Unit at Department of Experimental Medicine, “Sapienza” University Hospital of Rome and the Diabetes Unit at Siena University, and in the Obstetrics and Gynaecology out-patient Unit of Policlinico Umberto I, “Sapienza” University Hospital of Rome.

Women were screened for GDM according to current guidelines [61]. The enrollment was performed at third trimester of gestation. The exclusion criteria were as follows: non-Caucasian ethnic group; BMI ≥ 35 Kg/m^2^ before pregnancy; pre-pregnancy impaired fasting glucose (glycemia values 100–125 mg/dL); induced or multiple pregnancy; inflammatory, infectious and autoimmune diseases; polycystic ovarian syndrome; steroid therapy; alcohol and drug abuse; psychiatric diseases. At enrollment, a detailed clinical evaluation was performed, including medical history recording and anthropometric/vital parameters assessment (blood pressure, heart rate, weight, BMI,). At third trimester, fetal ultrasound parameters were measured. Venous blood samples were collected to assess laboratory parameters and miRNA expression. All subjects gave their informed consent for inclusion before they participated in the study. The study was conducted in accordance with the Declaration of Helsinki, and the protocol was approved by the Hospital Ethics Committee of “Sapienza” University of Rome (Project identification code 397-3435, date of approval 18 December 2014).

### 4.2. Blood Samples Processing

Blood samples were collected and stored in BD Vacutainer K2-EDTA tubes and processed within 2 h. Plasma was obtained by using a standardized operating procedure consisting of centrifugation at 1300× *g* for 10 min at room temperature (RT), at 1200× *g* for 20 min at RT and at 10,000× *g* for 30 min at RT. Plasma samples were stored at −80 °C until further use.

Unless otherwise stated, commercially available products were used according to the manufacturer’s instructions/protocols.

### 4.3. EVs Isolation and Characterisation

EVs were obtained from 500 µL of plasma using ExoQuick Plasma prep and Exosome precipitation kit (Cat #EXOQ5TM-1, System Biosciences, Palo Alto, CA, USA) following the manufacturer’s instructions. Briefly, plasma samples were precleared with thrombin and EVs were precipitated by adding the Exoquick Exosome Precipitation Solution. Precipitated EVs were re-suspended in 200 μL of RNase free H_2_O and checked for purity with western blotting.

For western blotting analysis, EVs were lysed with RIPA buffer (50 mM Tris-HCl pH7.6, 0.5% Sodium deoxycholate, 1% NP40, 0.1% SDS, 140 mM NaCl, 5 mM EDTA pH8, 100 mM NaF, 2 mM Na_4_P_2_O_7_·H_2_O) and Protease Inhibitor Cocktail (Sigma-Aldrich, Saint Louis, MI, USA). Immunoblotting was performed using standard procedures (Ronci et al., Molecular Biosystems 2015). Primary antibodies: rabbit anti-Tsg101 (Atlas Antibodies, HPA006161, Sigma-Aldrich), rabbit anti-Hsp70 (Santa Cruz Biotechnology, sc-33575, Dallas, Texas, USA, rabbit anti-Calnexin (Enzo Life Sciences Ag, Lausen, Switzerland, No. ADI-SPA-865) and rabbit anti-CD63 (Bio-Rad, VPA00798, Hercules, CA, USA) were diluted in TBS-T with 5% of Non-Fat Dried Milk (NFDM) and incubated overnight at 4 °C, then incubated with HRP-conjugated secondary antibodies diluted in TBS-T with 5% of NFDM and incubated 1 h at RT. Finally, membranes were incubated with enhanced chemiluminescence (Advansta, CA, USA) and images were acquired with Azure Biosystems Imagers (Dublin, CA, USA).

### 4.4. RNA Extraction

RNA from total plasma and EVs was obtained by using the automated Maxwell RSC-Promega extractor, from the Maxwell RSC miRNA Plasma and Serum kit (CAT # AS1680, Promega), following manufacturer’s instructions.

For the high-throughput screening assay performed on the discovery cohort, the starting volume of plasma for RNA extraction was 500 μL, whereas for RT-qPCR analysis on the validation cohort, the starting volume of plasma was 250 μL.

Ath-miR-159a spike-in was added during RNA extraction to follow the technical quality of the extraction.

### 4.5. Discovery Cohort: MiRNA Profiling and Differential Expression Analysis

The multiplexed NanoString nCounter Human v3 miRNA expression assay (NanoString Technologies, Seattle, WA, USA) was used to profile 800 human miRNAs of the discovery cohort. Sample preparation involved a multiplexed annealing of the specific tags to their target miRNA, a ligation reaction, and an enzymatic purification to remove the unligated tags, according to manufacturer’s protocol. The product was then diluted, denatured, combined with Reporter and Capture CodeSet, and hybridized to the Target-Probe Complex. Complexes were then immobilized on the cartridge for data collection. Digital images were processed and the barcode counts were tabulated in a comma separated value (CSV) format.

MiRNA raw data were subjected to a first quality control evaluation and underwent background subtraction and normalization using the five most stable miRNAs [62] for total plasma and EVs, respectively, using the nSolver 4.0 Software (NanoString Technologies inc., Seattle, WA, USA). Normalized RCC files were then loaded and analyzed by Rosalind nCounter Data Analysis Software (San Diego, CA, USA). The resulted DE miRNAs were filtered using *p*-value < 0.05 and ±1.5-Fold change (FC), as threshold of significance.

### 4.6. Validation Cohort: RT-qPCR Analysis

For miRNA validation analysis, we prepared two different Custom Reverse Transcription Pools and Custom Pre-amplification Pools for total plasma and EVs, respectively, following manufacturing instructions. MiRNAs used in the pools are reported in Supplementary Table S1. To perform normalization, U6 and the spike-in ath-miR-159a were used. In brief, RT miRNA pools were made using TE buffer (#12090015, Thermo Fisher, Waltham, MA, USA) and reverse transcription was performed using TaqMan^®^ MiRNA Reverse Transcription Kit (#4366597, Thermo Fisher). Then, pre-amplifications were made using the TaqMan^®^ PreAmp Master Mix (#4488593, Thermo Fisher) and the miRNA Pre-Amp pool. For PCR detection, TaqMan^®^ Universal Master Mix II, no UNG (#4440041, Thermo Fisher) was used.

### 4.7. Bioinformatics and Statistical Analysis

DE miRNAs between GDM and NGT patients were subjected to pathways enrichment analysis by using Diana-mirPath v3.0 [63]. Validated target genes were used as input for enrichment disease analysis and the genes relevant in GDM-related pathways were used for the selection of miRNAs to be further analyzed in the patients’ validation cohort.

Validated miRNAs were put as input in FANTOM5 project to identify their cell of origin [64], and their experimentally validated target genes were downloaded from the Homo sapiens Release 8.0 [65] and intersected. The common target proteins and mRNAs tissue expression of the validated miRNAs were checked by using the Human protein atlas v.21.0 [66].

Continuous variables and categorical variables were expressed as mean ± standard deviation (SD) or frequencies, respectively. The Kolmogorov–Smirnov test was performed to test for normality. Independent sample t-test or Mann–Whitney U test were used to compare means between groups for normally distributed or not normally distributed continuous variables, respectively. Fisher’s exact test was performed to compare categorical variables between groups. The association between miRNA expression and other variables was tested with Spearman’s correlation analysis. A *p*-value < 0.05 was considered statistically significant. Statistical analysis was performed with IBM SPSS Statistics software version 23 (Chicago, IL, USA).

Unpaired t-test was performed using GraphPad Prism software version 6.0 (La Jolla, CA, USA) to define the DE miRNAs of the validation cohort. *p* values < 0.05 were considered statistically significant.

## Figures and Tables

**Table 1:** Clinical and biochemical parameters of the discovery cohort.

	NGT	GDM	*p*-Value	
	(*n* = 3)	(*n* = 3)		
Age (years)	34.3 ± 3.1	34.7 ± 4.9	0.93	
Gestational week (*n*)	37.3 ± 1.2	36.6 ± 1.2	0.52	
Pre-pregnancy BMI (Kg/m²)	22.1 ± 0.5	26.3 ± 4.6	0.20	
Weight increase (kg)	11.6 ± 7.0	7.0 ± 4.3	0.15	
3rd trimester BMI (Kg/m²)	26.4 ± 1.1	27.0 ± 3.7	0.12	
Nulliparity (*n*)	1	1	-	
Insulin therapy (*n*)	-	0	-	
FPG (mg/dL)	70.9 ± 11.2	92.3 ± 15.4	0.12	
HbA1c (%)	5.3 ± 0.2	5.5 ± 0.3	0.53	
Foetal US Parameters				
Gestational week (*n)*	34.0 ± 3.0	35.0 ± 2.6	0.69	
AC (mm)	315.0 ± 23.6	298 ± 66.2	0.70	
HC (mm)	297.3 ± 30.9	305.7 ± 28.6	0.75	
Bi-parietal diameter (mm)	86.6 ± 8.7	89.5 ± 6.6	0.68	
Femur length (mm)	66.7 ± 7.0	68.2 ± 7.9	0.82	
Humerus length (mm)	57.0 ± 5.3	62.9 ± 1.5	0.23	
Estimated foetal weight (g)	2625 ± 639	2817 ± 736	0.75	

BMI: body mass index; FPG: fasting plasma glucose; HbA1c: glycated haemoglobin; US: ultrasound; AC: abdominal circumference; HC: head circumference. Data are expressed as mean ± standard deviation or frequency.

**Table 2:** Clinical and biochemical parameters of the validation cohort.

	NGT	GDM	*p*-Value
	(*n* = 12)	(*n* = 12)	
Age (years)	34.9 ± 5.1	36.4 ± 4.6	0.46
BMI (Kg/m²)	22.4 ± 3.2	25.8 ± 3.8	0.03 *
FPG (mg/dL)	68.5 ± 5.1	78.4 ± 6.8	0.002 *
Total cholesterol (mg/dL)	251.4 ± 80.1	276.7 ± 60.7	0.44
LDL-c (mg/dL)	132.6 ± 54.2	158.8 ± 59.5	0.34
HDL-c (mg/dL)	77.4 ± 14.8	76.6 ± 12.0	0.90
Triglycerides (mg/dL)	202.0 ± 81.9	207.0 ± 48.6	0.88
Creatinine (mg/dL)	0.53 ± 0.07	0.52 ± 0.08	0.75
Foetal US and Neonatal Parameters			
AC (mm)	288.4 ± 18.3	289.8 ± 14.8	0.90
HC (mm)	307.5 ± 18.5	303.4 ± 7.9	0.63
Femur length (mm)	63.3 ± 4.5	62.7 ± 3.8	0.75
Estimated foetal weight (g)	2133.8 ± 407.7	2101.0 ± 276.4	0.84
Birth weight (g)	3138.1 ± 242.2	3250.9 ± 452.3	0.54

BMI: body mass index; FPG: fasting plasma glucose; LDL-c: LDL cholesterol; HDL-c: HDL cholesterol; US: ultrasound; AC: abdominal circumference; HC: head circumference; * *p* < 0.05. Data are expressed as mean ± standard deviation.

**Table 3:** Total plasma and extracellular vesicles (EVs) differentially expressed (DE) miRNAs of the discovery cohort involved in GDM-related pathways.

Site	DE miRNA
**Total Plasma**	hsa-miR-222-3p
	hsa-miR-302d-3p
	hsa-miR-382-5p
	hsa-miR-409-3p
	hsa-miR-432-5p
	hsa-miR-625-5p
	hsa-miR-941
**EVs**	hsa-miR-16-5p
	let-7b-5p
	hsa-miR-106a-5p
	hsa-miR-17-5p
	hsa-miR-19b-3p
	hsa-miR-144-3p
	hsa-miR-20a-5p
	hsa-miR-20b-5p
	hsa-miR-93-5p
	hsa-miR-19a-5p
	hsa-miR-26b-5p
	hsa-miR-155-5p

**Table 4:** Overview of the three common miR-222-3p and miR-409-3p validated target genes with a role in T2D.

Official Symbol	Official Full Name	Sequence Accession ID	Function	References
MGMT	O-6-Methylguanine-DNA Methyltransferase	NM_002412.4	MGMT activity is lower in leukocytes derived from type 1 and type 2 diabetic subjects potentially contributing to mellitus diabetes pathogenesis	Akçai et al. Diabetes Res and Clinical Practice 2003 [45]
PPP2R2A	Serine/threonine-protein phosphatase 2A 55 kDa regulatory subunit B alpha isoform	NM_001177591.1	Low levels of PPP2R2A protein are responsible of a reduced insulin-induced phosphorylation of AKT protein in vivo and in vitro	Goldsworthy et al. Diabetes 2016 [46]
RECK	Reversion Inducing Cysteine Rich Protein With Kazal Motifs	NM_021111.2	RECK is down-regulated in diabetic kidneys and the SGLT2 inhibitor Empagliflozin partially restored its expression both in vivo and in vitro	Aroor et al. Cardiovasc Diabetol 2018 [47]

## Data Availability

The data presented in this study are available on request from the corresponding author.

## Supplementary Materials

The following supporting information can be downloaded at: www.mdpi.com/article/10.3390/ijms23084276/s1.
