# Supplementary material for: Identification and Validation of miR-222-3p and miR-409-3p as Plasma Biomarkers in Gestational Diabetes Mellitus Sharing Validated Target Genes Involved in Metabolic Homeostasis
Source: Int J Mol Sci. 2022 Apr 12;23(8):4276. doi: 10.3390/ijms23084276 (PMC9028517; doi:10.3390/ijms23084276)
Supplement: Supplementary file 1 [file ijms-23-04276-s001.zip › Supplementary Figure S1.pdf]

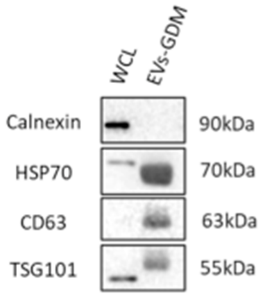

Figure S1. Characterisation of extracellular vesicles (EVs) plasma derived. Western blotting analysis of Calnexin, checked as negative marker; Tsg101, CD63 and Hsp70 EVs-enriched markers. WCL, whole cell lysate; EVs-GDM, gestational diabetes mellitus (GDM) plasma-derived extracellular vesicles (EVs).
